# Protective Role of Physical Exercise and Exercise‐Induced FNDC5/Irisin in Combating Diabetes‐Related Cognitive Impairment and Autonomic and Peripheral Neuropathies: A Comprehensive Review

**DOI:** 10.1155/jdr/5552311

**Published:** 2026-02-18

**Authors:** Sepideh Poshtdar, Hosein Ataei-Goujani, Amirali Ahrabi

**Affiliations:** ^1^ School of Medicine, Tehran University of Medical Sciences, Tehran, Iran, tums.ac.ir; ^2^ Eye Research Center, Farabi Eye Hospital, Tehran University of Medical Sciences, Tehran, Iran, tums.ac.ir; ^3^ Student Research Committee, Shahrekord University of Medical Sciences, Shahrekord, Iran, skums.ac.ir; ^4^ Sina Trauma and Surgery Research Center, Tehran University of Medical Sciences, Tehran, Iran, tums.ac.ir

**Keywords:** aerobic exercise, cognitive impairment, diabetes mellitus, diabetic neuropathy, FNDC5, irisin, resistance training

## Abstract

The rising prevalence of diabetes has been closely linked to increased mortality and morbidity, particularly as its complications become more widespread. Among the most challenging of these complications are cognitive impairments and diabetic neuropathies, which are neurodegenerative conditions that significantly diminish the quality of life in the diabetic population. Beyond its well‐established benefits for improving glycemic control and preventing cardiovascular complications, physical exercise has also been shown to mitigate central and peripheral neurodegenerative complications of diabetes, including cognitive decline, peripheral neuropathies, and autonomic neuropathies. Emerging research suggests that fibronectin type III domain‐containing protein 5 (FNDC5), later converted into irisin, an exercise‐induced myokine, plays a key role in mediating these protective effects. This narrative review thoroughly examines the protective impact of various forms of physical exercise—including aerobic, resistance, and multimodal training—on diabetic neuropathies and cognitive decline, while also exploring the involvement of FNDC5/irisin in these beneficial outcomes across experimental animal studies, cross‐sectional analyses, and controlled trials.

## 1. Introduction

According to the International Diabetes Federation′s (IDF) 11th Edition of the *IDF Diabetes Atlas* (2025), approximately 589 million adults aged 20–79 years, equivalent to 11.1% of the global adult population (roughly 1 in 9), are living with diabetes. Alarmingly, nearly 43% of these individuals (over 250 million adults) remain undiagnosed, many of whom may already be at risk of developing diabetes‐related complications. Looking ahead, the IDF projects that by 2050, the global number of adults with diabetes will rise to 853 million (about 1 in 8 adults worldwide), underscoring the growing magnitude of this public health challenge [1]. As the prevalence of diabetes continues to escalate, its complications are rising concurrently as well, presenting major challenges for diabetes management [2]. Diabetes is associated with significant complications related to the central nervous system, the peripheral nervous system, and the autonomic nervous system (ANS), including cognitive impairment, diabetic peripheral neuropathy (DPN), and cardiac autonomic neuropathy (CAN) [3–5].

Diabetic patients are also at a twofold risk for developing dementia, with approximately 2.5% of diabetic patients currently suffering from this condition [5, 6]. In recent years, dementia‐related mortality among diabetic patients has surpassed the heart disease–related causes and is considered one of the leading causes of death among diabetic patients [7]. Besides the association of diabetes with the development of dementia, about 14% of diabetic patients are faced with a condition of lesser severe cognitive impairment, known as mild cognitive impairment (MCI) [5]. Diabetes can impair different cognitive domains, mostly executive function and processing speed [8]. Moreover, diabetic cognitive impairment can significantly deteriorate patients′ self‐glycemic care, leading to life‐threatening outcomes such as hypoglycemia, ketoacidosis, and hyperosmotic coma [9]. Up to this point, there is no fully recognized pathophysiological mechanism responsible for the development of cognitive impairment in diabetic patients; however, it is speculated that a combination of vascular and neurodegenerative processes is involved in this setting [10]. Recognizing the need for further research in this area, the National Institutes of Health (NIH) Diabetes Mellitus Interagency Coordinating Committee (DMICC) has emphasized the importance of studying diabetes‐associated cognitive impairment in their strategic plan [11].

DPN is considered the most common complication of diabetes [12]. DPN is closely linked with increased sleep disturbances, heightened anxiety, and a significant decline in quality of life [13]. Patients with DPN complain of a sense of burning, prickling, deep aching, or sharp sensations [14]. However, some of the patients may lose the ability to sense pain and temperature [15]. Patients with the painful form of DPN are mostly managed with anticonvulsants. However, about 50% of cases are resistant to this therapy; therefore, they should be managed with opioids [16].

Besides the previously discussed diabetic complications, CAN represents another form of diabetic‐associated autonomic dysfunction, strongly associated with increased mortality [4]. Both Type 1 and Type 2 diabetes (T1D and T2D) increase the risk of developing CAN [17]. The prevalence of CAN among diabetic patients ranges widely, from 2.5% to as high as 90%, depending on the disease status of the patient [18]. CAN is commonly underdiagnosed due to many diabetic patients being asymptomatic. However, presyncope, syncope, palpitation, and dizziness might develop in the late stages of CAN [19]. Heart rate variability (HRV) can be considered a valuable means of early diagnosis of CAN among asymptomatic patients, as it was applied in some of the studies [14].

Physical exercise, an important element of a healthy lifestyle, could have a significant role in the management of diabetes, as it is regarded as a foundational element in the first‐line treatment of T2D [20]. Besides its footprint in glycemic control, cardiovascular health, and blood pressure regulation [21], physical activity could be helpful in regard to mitigating diabetic complications such as CAN [22], DPN [23], and diabetes‐associated cognitive impairment [24]. Therefore, the American College of Sports Medicine and the Diabetes Association (ACSM) recommends moderate to vigorous intensity aerobic exercises, for at least 150 min per week (30 min, 5 days/week), next to resistance‐type exercises twice or three times a week [25, 26]. Irisin/FNDC5 (fibronectin type III domain‐containing protein 5), a myokine initially discovered by Boström et al. in 2012 [27], has garnered significant attention for its potential role in improving insulin resistance, since its discovery [28]. It has been established that exercise can elevate circulating irisin levels, contributing to its beneficial effects on vascular and metabolic health [29, 30]. In some studies, the role of irisin in diabetic complications was evaluated, with the potential association of this myokine in the mitigation of diabetic neuropathies [31–34]. However, the importance of this exercise‐induced myokine needs to be further studied. Therefore, in this narrative review, we aim to explore the role of physical activity—particularly through the production of FNDC5/irisin—in mitigating diabetes‐induced cognitive impairment, autonomic neuropathy, and peripheral neuropathy. Additionally, we will investigate the neuroprotective properties of FNDC5/irisin as a novel exercise‐induced myokine.

## 2. Methodological Approach

### 2.1. Review Type and Scope

This narrative review integrates evidence from clinical and preclinical studies on the effects of structured exercise on diabetes‐related cognitive impairment, DPN, and CAN, with particular focus on the role of irisin, the cleavage product of FNDC5. Terminology is applied consistently throughout the manuscript to avoid ambiguity.

### 2.2. Literature Search

We conducted a structured search of PubMed, Scopus, and Web of Science for studies published up to July 2025. The search combined controlled vocabulary (e.g., MeSH terms) and free‐text keywords related to four main domains: (i) exercise and physical activity (“exercise,” “aerobic training,” “resistance training,” “strength training,” “endurance,” “high‐intensity interval training,” “HIIT,” “multimodal exercise,” “physical activity”); (ii) diabetes (“type 1 diabetes,” “type 2 diabetes,” “diabetic,” “streptozotocin,” “diet‐induced diabetes,” “experimental diabetes models”); (iii) neurological outcomes (“cognition,” “memory,” “executive function,” “learning,” “diabetic neuropathy,” “peripheral neuropathy,” “small fiber neuropathy,” “autonomic neuropathy,” “cardiac autonomic neuropathy,” “CAN,” “DPN,” “nerve conduction,” “heart rate variability”); and (iv) molecular mediators (“irisin,” “FNDC5,” “myokine,” “neurotrophic factor”).

Boolean operators were used to combine terms, for example, (“exercise” OR “physical activity” OR “training”) AND (“diabetes” OR “type 1 diabetes” OR “type 2 diabetes”) AND (“cognition” OR “neuropathy” OR “autonomic dysfunction” OR “irisin” OR “FNDC5”). Filters for language (English) were applied to refine results. In addition to database searching, the reference lists of relevant articles and recent reviews were hand‐checked to identify additional publications that might not have appeared in the electronic search. The overall approach was designed to ensure that both interventional and mechanistic studies were captured, spanning clinical, experimental, and translational research.

### 2.3. Eligibility Criteria

Studies were included if they (i) enrolled adults with T1D or T2D or used established animal models of diabetes; (ii) involved structured exercise interventions or assessed habitual physical activity; and (iii) reported outcomes relevant to cognition, DPN, CAN, or irisin. Studies were excluded if they were editorials, letters, conference abstracts, or non–peer‐reviewed articles; did not assess the outcomes of interest; or were published in languages other than English without available translation. In clinical studies, participants were generally required to have a confirmed diagnosis of diabetes, while animal studies typically used streptozotocin (STZ)‐induced or diet‐induced diabetes models.

### 2.4. Study Design and Randomization

Study designs were recorded as described by the authors. Randomized controlled trials (RCTs) were distinguished from nonrandomized trials, observational studies, and experimental models. When authors specified randomization procedures or blinding of participants or assessors, these details were noted. This allowed randomized and nonrandomized evidence to be considered separately where appropriate, while preserving the methodological context provided in the original reports.

### 2.5. Data Extraction

From each study, we summarized population characteristics (age, diabetes type, and sample size), details of the exercise intervention (modality, frequency, session duration, intervention length, and reported intensity), comparator groups where relevant, and the principal outcomes. For animal studies, we also extracted information on the type of diabetes model, exercise paradigm, and molecular or histological assessments. Assay type (i.e., ELISA for irisin) or testing methods were described when specified by the study authors.

### 2.6. Outcome Measures

Cognitive function in human studies was most often assessed using the Mini‐Mental State Examination (MMSE), Montreal Cognitive Assessment (MoCA), Stroop test, or Trail Making Test, while animal studies relied on tasks such as the Morris Water Maze (MWM) and passive avoidance. DPN was evaluated using validated scales including the Michigan Neuropathy Screening Instrument (MNSI), Michigan Diabetic Neuropathy Score (MDNS), and Toronto Clinical Scoring System, together with nerve conduction studies, intraepidermal nerve fiber density (IENFD) from skin biopsy, and quantitative sensory testing. CAN was primarily assessed by HRV indices such as standard deviation of R‐R intervals (SDNN) and root mean square of successive differences between normal heartbeats (rMSSD), with frequency–domain measures and baroreflex sensitivity reported in some studies. Neuropathic pain and quality of life were measured with disease‐specific questionnaires when available. Irisin was quantified either as circulating concentrations by immunoassay or as tissue expression among some of the preclinical studies. To strengthen interpretability, we reported effect estimates (e.g., mean or percent changes and standardized mean differences [SMDs]) together with 95% confidence intervals whenever available. When original studies did not provide confidence intervals or standardized effect sizes, the magnitude and direction of change were described to complement *p* values, ensuring that statistical significance is interpreted within its clinical or biological context.

### 2.7. Integration of Evidence

Because of the diversity of study designs, interventions, and outcome measures, findings were presented narratively rather than statistically pooled. Results are organized by outcome domain (cognition, DPN, and CAN) and by exercise modality, with human and preclinical evidence described separately where relevant.

## 3. The Beneficial Role of Exercise Against Diabetic Complications

Diabetes, being a concerning epidemic challenge, affects a wide range of people of all ethnicities worldwide, while burdening global health, economy, and healthcare systems to a great magnitude, throughout the increase of morbidity and mortality rates, especially in association with the development of chronic complications in the course of the disease [35, 36]. Diabetes has been associated with a wide range of complications, including microvascular and macrovascular complications. Diabetes complications, such as neuropathy, retinopathy, nephropathy, diabetic foot ulcers, and myocardial infarction, have been confirmed to significantly affect the quality of life of diabetic patients. Patients suffering from chronic diabetes mellitus (DM) complications struggle with physical functioning and daily life activities [36, 37]. The immense burdens of diabetic neuropathy in association with physical and mental health have been thoroughly reviewed in the literature as well [38].

Cognitive decline is increasingly recognized as a complication of diabetes, often beginning with executive dysfunction and progressing to MCI or dementia. This process involves multiple neurodegenerative mechanisms, including oxidative stress, chronic hyperglycemia, microvascular injury, impaired insulin signaling, and reduced neurogenesis and synaptic plasticity [39, 40]. Emerging work has shown that exercise‐induced upregulation of PGC‐1*α* and FNDC5/irisin counters many of these pathways by enhancing BDNF‐mediated neurogenesis and reducing oxidative and inflammatory stress in the hippocampus [41, 42].

The ACSM defines aerobic or endurance exercises as activities that are continuous and rhythmic and involve large muscle groups. Common examples of aerobic physical activities include running and swimming [43]. In addition to the association of aerobic exercises with glycemic control, insulin sensitivity, and cardiovascular system strength, aerobic exercises might be helpful in the amelioration of diabetic neuropathy [44, 45]. On the other hand, resistance training is described as “a form of physical activity that is designed to improve muscle fitness by exercising a muscle or muscle group against external resistance” [46]. Resistance training has also been proven to improve glycemic control and diabetes complications [21]. In light of the beneficial effects of both exercise types in managing diabetes and its complications, some studies explored multimodal or combined physical activity as candidates for mitigating diabetic complications [6, 47, 48]. Therefore, due to the high burden of diabetic neuropathy and diabetic cognitive impairment, as degenerative diabetic complications, in the following sections of our study, we aim to thoroughly review the possible protective and ameliorative effects of different types of physical activity in the mitigation of these complications.

### 3.1. The Effects of Exercise Against Diabetic Cognitive Impairment

Diabetes substantially increases the risk of cognitive impairment, from MCI to dementia, with a particularly strong association with Alzheimer‐like changes. These impairments are linked to hyperglycemia, insulin resistance, neuroinflammation, and cerebral microvascular dysfunction [49, 50]. Recent studies have highlighted the role of amyloid (A*β*), tau, and neurofilament light (NFL) proteins as markers of neurodegeneration in diabetes‐related cognitive impairment. The plasma A*β*42/40 ratio, p‐tau181, and NFL concentrations are elevated in diabetic individuals with cognitive decline. These markers are implicated in the development of Alzheimer‐like pathologies. Exercise, particularly through the increase in irisin, has been shown to modulate these pathways. Irisin has been suggested to reduce amyloid burden and tau hyperphosphorylation, which may contribute to the cognitive improvements observed following physical exercise in diabetic patients [51–54].

Both T1D and T2D are associated with cognitive decline and dementia, impacting executive function, working memory, attention, and processing speed in affected individuals [55].

**Figure 1:**
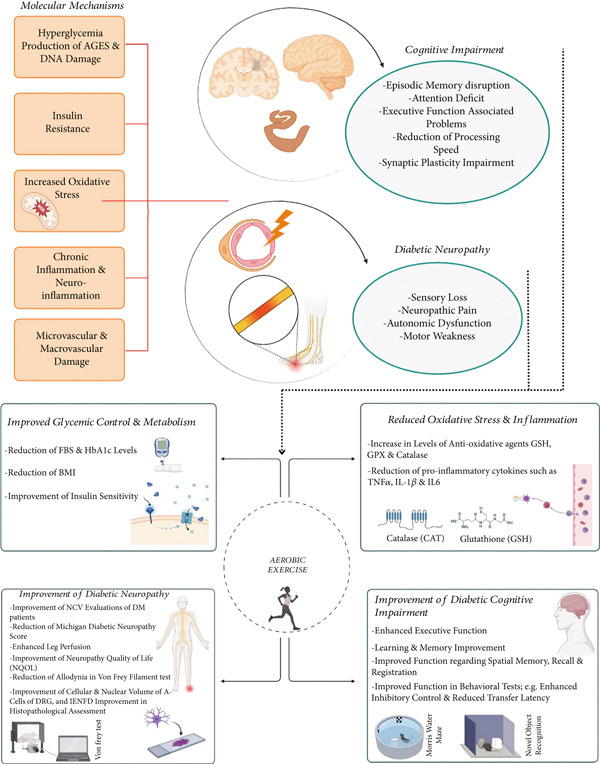
Key pathways in diabetes‐related cognitive impairment and neuropathy and the protective role of aerobic exercise. A summary of molecular mechanisms (hyperglycemia, oxidative stress, and inflammation) driving diabetes complications, alongside exercise‐induced benefits such as improved metabolism, reduced neuroinflammation, and enhanced neuronal function. Evidence from the reviewed studies supports these effects.

MCI, characterized as an intermediate state of cognitive decline between normal aging‐related cognitive decline and dementia, can be associated with impairment of language, memory, thinking, and judgment processes [55, 56]. Given the increasing incidence of diabetes‐related MCI and the lack of effective pharmacologic therapies, physical exercise has emerged as a promising intervention. Through metabolic improvements, reduction of neuroinflammation, and enhancement of synaptic plasticity, exercise may slow the progression from MCI to dementia in diabetic patients. Figure 1 outlines the proposed key mechanistic pathways.

To begin, several experimental and trial studies, detailed in Table 1, have investigated the beneficial effects of physical activity in the amelioration of diabetes‐associated cognitive impairment [74]. For example, in human subjects, Molina‐Sotomayor et al. explored the effects of aerobic walking physical activity on 57 diabetic women with MCI, in a longitudinal study for 6 months. A significant improvement was observed in various aspects of the MMSE test scores, including time and spatial orientation, registration, calculation, recall, and language. The most substantial enhancements were observed among the registration and recall dimensions [57]. Additionally, in a randomized controlled study, individuals with glucose intolerance and normal cognitive status exhibited a significant increase in executive function, encompassing attention, cognitive flexibility, and working memory, as well as an improvement in insulin sensitivity and cardiovascular function, following 6 months of vigorous intensity aerobic exercise, compared to a control group engaged in stretching activities [58]. In a randomized trial of 328 adults with T2D and MCI, Chen et al. compared tai chi chuan—a moderate‐intensity regimen integrating aerobic, balance, and meditative components—with fitness walking. Both interventions improved MoCA scores at 24 weeks, but at 36 weeks, tai chi produced superior gains in global cognition (mean between‐group difference 0.84 points, 95% CI 0.02–1.66, *p* = 0.046) [75]. The multimodal nature of tai chi, combining physical and cognitive engagement, may explain its stronger neuroprotective effects. Wang et al., in a clinical trial study on 82 T2D patients, confirmed that a year of aerobic training could significantly increase the hippocampal volume, while improving cognitive function, as evidenced by better results in MMSE and MoCA scores [70]. Moreover, observational evidence (*n* = 2646 older Chinese adults with T2D) also suggests exercise protects against cognitive decline and alleviates depression and sleep problems (coefficient = −0.6858, *p* < 0.001) [76]. While human studies can demonstrate the efficacy of such approaches, more insights into the mechanisms through which exercise exerts its effects on diabetes‐related cognitive impairments including the important outcomes from animal studies are reviewed here, as well. Moreover, in the Israel Diabetes and Cognitive Decline (IDCD) cohort, 367 cognitively normal T2D patients (mean age 72.4 years) were followed for 42 months. Physically active participants, as measured by the Minnesota Leisure Time Activity Questionnaire, had significantly lower rates of decline in global cognition, executive function, and working memory compared with inactive peers, though episodic memory and semantic categorization were unaffected [77]. This might suggest that habitual exercise preserves higher‐order cognitive domains most vulnerable to diabetes‐related decline. Furthermore, a meta‐analysis of five RCTs including a total of 738 T2D patients suggested that exercise markedly improved global cognitive function among these patients, regardless of the modality and duration of the intervention (SMD: 1.34, 95% CI: 0.23–2.44, *p* < 0.01) [74].

**Table 1:** A summary of studies investigating the beneficial effects of aerobic, resistance, or multimodal exercise regarding diabetic complications and cognitive impairment.

**List**	**Study design**	**Study subjects**	**Type and intensity of physical training**	**Duration of the study**	**Cognitive tests**	**Key findings**
Human studies/aerobic exercise						
Molina‐Sotomayor et al. [57]	Experimental and longitudinal study	Older women with Type 2 diabetes (with an experimental group [*n* = 57] and a control group [*n* = 52])	Walking‐based (aerobic) training	6 months	MMSE	In addition to improvement of HbA1c and BMI measures, cognitive functioning in all its dimensions (other than calculation and language) was enhanced significantly, with these outcomes being more prominent in the experimental group compared to controls.
Baker et al. [58]	A randomized controlled design	28 adults (57–83 years old) with glucose intolerance (according to 2‐h tolerance test criteria)	Supervised aerobic exercise sessions (in the experimental group) and stretching (control group)	6 months	‐Executive function (Trails B, task switching, Stroop, self‐ordered pointing test, and verbal fluency) ‐Memory performance (story recall, list learning)	In addition to improvement of insulin sensitivity and cardiorespiratory fitness, subjects undergoing 6 months of aerobic exercise had enhanced executive function.
Harveson et al. [59]	Randomized crossover design	94 untrained teenager participants (with a mean BMI of 22.57 ± 3.8)	Participants undergoing 30 min of aerobic or resistance exercises or no exercises	7 days (aerobic exercise protocol including 30 min of walking/jogging and resistance training protocol consisting of weight machines and exercises such as squats)	‐Stroop test including the dot, word, and color elements—TMT, Parts A and B	Both types of acute exercises led to enhanced cognition among untrained youth participants, as evidenced by better performance in ST. Aerobic exercise group also demonstrated better outcomes regarding Part B of the TMT compared to controls and resistance exercise groups.

Animal studies/aerobic exercise						
Mehta et al. [60]	Experimental animal study	STZ‐induced T2D rats	Aerobic exercise on running wheel	6 weeks, 5 days/week, 150 m/day (25–30 min)	‐Passive avoidance test ‐Elevated plus maze	Aerobic exercise led to improved insulin resistance and glycemic control (decreased FBS and HbA1c levels), elevated hippocampal GSH levels, CA1 and CA3 neuronal density, lowered acetylcholinesterase activity, and reduced measures of IL‐1*β*, TNF‐*α*, and MCP‐1 neuroinflammatory markers. Cognitive improvement was evident by increase of step‐down and reduction of transfer latency.
De Senna et al. [61]	Experimental animal study	STZ‐induced T1D rats	Treadmill running aerobic training	5 weeks (starting with a duration of 20 min/session, up to 60 min by the second week)	Place recognition (PR) test	Exercise leads to amelioration of diabetes‐induced spatial memory decline, including improvement of reduced relocated object exploration time, along with increase of GSH and GFAP‐positive astrocyte density.
Karimi et al. [62]	Experimental animal study	STZ‐induced T2D rats	Treadmill running and swimming aerobic exercise groups	2 months, 5 consecutive days/week; running at a speed of 25 m/min, for 1 h, swimming for 60 min/day	‐Step‐through latency in the retention test (STLr) ‐The time spent in the dark compartment (TDC)	Regular aerobic exercises led to enhancement of memory and learning which was impaired in diabetes, potentially via activation of antioxidative agents (increase of catalase and glutathione peroxidase [GPx] subsequent to exercise).

Human studies/resistance or combined training						
Yamamoto et al. [63]	Prospective RCT	60 elderly T2D patients	Resistance training using elastic bands or bodyweight at home	48 weeks	MMSE	Resistance exercise led to the significant improvement of cognitive impairment and knee extension strength compared to the nonexercising controls.
Furlano et al. [64]	Pilot RCT	Elderly patients (60–80 years old) with at least one of diabetes risk factors (i.e., prediabetes and obesity)	Participants allocated into resistance training group and stretching exercise group as controls	6 months (three times a week)	‐Stroop test; Condition C_B (for selective attention and response inhibition)—TMT (Part B_A) to measure task‐switching digit span test (for working memory) —Rey auditory verbal learning test	Improved neurocognition, as evidenced by enhanced attention, conflict resolution, and task‐switching skills along with enhanced brain activation patterns, was observed subsequent to resistance training.
Silveira‐Rodrigues et al. [10]	Pilot study (within‐subject counterbalanced trial)	11 T2D patients (63 ± 7 years old)	Moderate‐intensity acute aerobic (AER) and resistance exercise (RES) sessions	One session (treadmill walking for 40 min in AER and 3 sets of 10 repetitions for 8 strength exercises in RES)	‐Stroop color and word (SCW) task (for assessment of attention and inhibitory control) ‐Visual response time (RT)	Both types of exercises led to enhancement of incongruent‐SCW and RT_(best)_ among DM patients. However, BDNF was increased and reduced subsequent to AER and RES, respectively.
Callisaya et al. [6]	Pilot parallel RCT	50 T2D participants (50–75 years old)	Multimodal exercise program (a progressive aerobic and resistance training program [compared to a control group subjected to gentle movements])	6 months	Stroop test (ST), TMT, WAIS‐III, COWAT, Hopkins verbal learning test–revised (HVLT)	Multimodal exercise program led to improvements of global cognitive score, Stroop C‐D, Trails A‐B, DSC, Hopkins intermediate and recognition scores, COWAT‐word and Rey complex copy tests among diabetic patients.
Espeland et al. [65]	RCT	Sedentary nondemented elderly participants (70–89 years old), 415 of them with DM and 1061 without DM	A multimodal approach including moderate‐intensity walking, strength and balanced training, and flexibility	24 months	Modified Mini‐Mental State Exam (3MSE), WAIS III, Digit Symbol Coding (DSC) test, HVLT‐D	Multimodal exercise led to improved cognition of DM patients, especially regarding global cognitive function and delayed memory.
Stomby et al. [66]	Single‐blinded interventional controlled study	24 T2D patients randomized into Paleolithic diet and diet and exercise groups equally	High‐intensity supervised aerobic and resistance exercise (multimodal training)	12 weeks, 180 min/week	Episodic memory test (using a face‐name paired‐associate task)	Both interventional groups showed improved insulin sensitivity and weight loss, in addition to increase of functional responses and volume of the right hippocampus. Memory performance, however, remained unaffected.
Wang et al. [67]	Within‐subject cross‐sectional study	30 hospitalized T2D patient aged 45–70 years	30 min of AE, RE, and ICE exercises with 10 min of warm‐up and stretching	Each exercise regimen was performed on a separate day with a 48‐h time interval between each modality	‐Stroop test, more‐odd shifting, and 2‐back tests for assessment of executive function (for assessment of executive function) ‐Multi‐channel fNIRS system (for monitoring local cerebral oxygenated hemoglobin concentration changes)	‐ICE was demonstrated to be the most superior exercise modality regarding executive function improvement among T2D patients. ‐There is a potential link between activation of specific brain areas and different dimensions of cognitive function, suggesting a synergistic mechanism between them.
Ghahfarrokhi et al. [71]	Proof‐of‐concept pilot study	48 T2D participants	T2D patients were allocated equally into high‐intensity low‐volume (HIFT) and low‐intensity high‐volume (LIFT) and control groups	6 weeks/5 sessions each week between 40 and 45 min	‐Symbol Digit Modalities Test (SDMT) ‐California Verbal‐Learning Test Second Edition (CVLT‐II) ‐Brief Visuospatial Memory Test‐Revised (BVMT‐R)	Although both exercise modalities led to significant enhancement of glycemic control (improvement of fasting glucose, HOMA‐IR, and HbA1c), as well as Stroop and MMSE scores, these outcomes were more prominent subsequent to HIFT modality.

Animal studies/resistance exercise						
De Sousa et al. [72]	Experimental animal study	Dexamethasone‐induced T2D rats	High‐intensity resistance training (HIRT)	4 weeks	‐The novel object recognition task ‐The open field task (for assessment of locomotor activity and anxious behavior)	Despite no significant differences in locomotor activity and anxious behavior, exercising T2D rats showed improved glucose levels, attenuated hippocampal neuronal death, and maintained cognition compared to nonexercising diabetic rats facing cognitive decline.
Zarrinkalam et al. [73]	Experimental animal study	STZ‐induced T1D rats subjected to exercise (DR rats) or a combination of exercise and natural antioxidants (DRH rats)	Resistance training in the form of ladder climbing	10 weeks (5 days/week)	‐Morris Water Maze (MWM) test ‐Shuttle box tests for assessment of learning and memory function	‐Resistance training led to reduction of mean of total escape latency aspect of MWM test which was increased subsequent to DM, with this reduction being more significant in the DRH group. ‐TDC dimension of shuttle box test was significantly lowered among exercising diabetic rats compared to nonexercising DM rats or the ones only under antioxidant therapy.

Multiple preclinical studies have proven the effect of aerobic exercises on the different cognitive functions and brain structures associated with them [60–62, 78]. For instance, Mehta et al. investigated the effects of a 6‐week regular wheel running exercise on the contextual memory in diabetic rats. This exercise regimen led to increased recall of pain‐related emotions in the passive avoidance test, as well as improvements in contextual memory compared to sedentary diabetic rats [60]. The hippocampal regions CA1 and CA3, which are crucial for normal cognition, as their substantial role in the acquisition of context‐dependent extinction was established in previous studies [79], were also positively influenced subsequent to aerobic exercises. Increase of brain‐derived neurotrophic factor (BDNF) in CA3 hippocampal neurons, suppression of NOX2‐induced oxidative stress, and subsequent production of reactive oxygen species (ROS) leading to attenuation of CA1 region neuronal injury, as well as increased neuronal density of hippocampal CA1 and CA3 regions, were some of the positive effects of aerobic exercise observed among diabetic animal models [60, 80, 81]. Moreover, aerobic exercise, in the form of treadmill running, also mitigated spatial memory deficit in T1D rats, possibly through astrocytic ramification and increasing the density of glial fibrillary acidic protein (GFAP)–positive astrocytes [61]. Further research on regular aerobic exercises among diabetic rats revealed that both treadmill and swimming exercises were effective in the amelioration of the pathological effects of STZ‐induced impairment in passive avoidance learning ability. However, no significant differences were observed between these two types of aerobic exercises [62]. Molecular investigations have further elucidated the remarkable outcomes of aerobic exercises in animal models, confirming the role of aerobic treadmill exercise in reducing the expression of proapoptotic proteins (i.e., BAX and Caspase‐3) and subsequently mitigating the neuronal number decline in CA1 and CA3 regions of the hippocampus among diabetic female rats [78].

While the aforementioned studies in humans and preclinical models explored aerobic exercise, resistance training has also demonstrated promising outcomes regarding the improvement of cognitive function in diabetic individuals, as well [10, 59, 63, 64]. A recent systematic review [82] enrolling individuals with T2D, insulin resistance, or impaired glucose tolerance, with a total of 2289 participants from six studies, highlighted the potential benefits of resistance training, including improvements regarding insulin sensitivity, glycemic control, and inflammation. High‐intensity resistance exercise, in particular, may be better tolerated by individuals with multiple comorbidities compared to moderate‐to‐high intensity aerobic exercise, and it has been associated with positive effects on neurobiological factors such as BDNF and insulin‐like growth factor 1 (IGF‐1). Supporting this, an investigation comparing the effects of 30 min of resistance training to aerobic training in terms of cognitive benefits among 94 young untrained individuals, sampled from a public high school in Southwestern United States, confirmed that resistance training yielded superior results [59]. It seems that expanding the body of evidence in this area as well as carrying out high‐quality trials focusing on the impact of resistance training on cognitive impairment in diabetes is warranted [74, 82]. Notably, a recent study by Yamamoto et al. demonstrated that 12 months of resistance physical activity prevented cognitive decline [63]. Additionally, a study conducted on elderly individuals (aging 60–80 years old) at risk for diabetes due to risk factors such as obesity confirmed that 26 weeks of progressive resistance training improved various cognitive domains, including task‐switching performance, selective attention, and response inhibition, compared to a control group engaged in balance and tone exercises [64]. In addition, in line with the significant effects of chronic exercise, acute exercises seem to be helpful in improving impaired cognition of diabetic individuals, as well [10].

Providing more insights into the mechanistic factors underlying these effects, preclinical studies of resistance training have shown substantial outcomes in mitigating cognitive dysfunction induced by diabetes. Using a resistance training model resembling human squats, the Tamaki model, De Sousa et al. demonstrated that 4 weeks of high‐intensity resistance training could alleviate cognitive dysfunction subsequent to diabetes induction. This intervention also led to a significant reduction in hippocampal neural apoptosis and improvement of insulin resistance [72]. Another study found that 10 weeks of resistance training improved performance in the MWM test, as well as enhanced long‐term memory in the passive avoidance learning test [73]. To further elucidate the mechanisms involved concerning these beneficial effects, it can be stated that diabetes and hyperglycemia result in increased oxidative stress, neuroinflammation, death of cortical neurons, and subsequent behavioral deficits. Physical exercise, however, increases the total antioxidant capacity, while decreasing lipid peroxidation and improving neurogenesis, synaptic plasticity, and brain cell proliferation. The aforementioned effects may be accountable for the beneficial roles of physical exercise, especially regarding the improvement of spatial and passive avoidance learning and memory [73, 83]. According to a recent systematic review and meta‐analysis on DM animal models, including 17 studies, physical exercise led to a significant reduction of fasting glucose levels and escape latency in the MWM test. Additionally, it improved swimming speed, the number of platform crossings, and percentage of time spent in the target quadrant. This meta‐analysis also demonstrated a reduction of oxidative stress, A*β* deposition, neuroinflammation, and apoptosis, as well as enhancement of neurogenesis and synaptic function [84].

Several studies have explored the effects of combining aerobic and resistance training, characterized as multimodal exercise interventions. In a 6‐month pilot RCT, a multimodal exercise program incorporating progressive resistance training and aerobic exercise was found to improve cognitive function of T2D patients in a battery of neuropsychological tests (i.e., global cognitive score, Stroop C‐D, Trails A‐B, DSC). In addition, brain MRI study shows that present multimodal intervention was able to increase hippocampal and total brain volumes, in addition to mitigating the decline in white matter volume over the study period [6]. In a study involving a large cohort of participants (a total of 1476 participants), between 70 and 89 years old, with multiple comorbidities, such as hypertension, cardiovascular diseases, and diabetes (415 patients), 24 months of combined physical activity demonstrated improvement of global cognitive function and delayed memory in diabetic patients. Contrary to diabetic participants, patients with other comorbidities did not show improving effects. This difference in outcomes may be attributable to varying underlying pathophysiological mechanisms [65]. In line with the previous study, a 12‐week combined exercise study assessed the effects of physical activity in combination with a specific diet, the Paleolithic diet, in comparison to a diet‐only group among T2D patients. They evaluated the changes observed in fMRI and brain function of the patients. The results indicated an increase in hippocampal volume in patients who engaged in physical activity, despite no significant changes in memory performance [66]. Furthermore, Wang et al. [67] aimed to assess how acute bouts of different forms of exercise could affect executive function and cerebral hemodynamics among T2D patients. In this cross‐sectional study on 30 hospitalized T2D patients, they indicated that integrated concurrent exercise (ICE) leads to more notable outcomes in improving executive function compared to other modalities. Additionally, concurrent activation of dorsolateral prefrontal cortex (DLPFC), the frontal polar (FPA) and orbitofrontal cortex (OFC) blood flow was observed subsequent to executive function enhancement in ICE. Moreover, inhibitory function improvement and synchronous DLPFC and FPA activation, in addition to pars triangularis Broca′s area HbO2 concentration increment and improvements of refresh function, were also observed subsequent to resistance and aerobic exercises, respectively. Additionally, in a recent proof‐of‐concept pilot study with 48 T2D participants, aged 67.5 ± 5.8 years, Ghahfarrokhi et al. [71] aimed to investigate how different intensities of functional training affect diabetic cognitive decline. They applied a protocol of exercise, combining the endurance, balance, and strength exercises, at two different volumes and intensities. Both training modes significantly improved Stroop and MMSE scores. High‐intensity low‐volume (HIFT) approach, however, was confirmed to be more effective in improving physical, biochemical, and cognitive function. Moreover, a recent meta‐analysis by Lu et al., including eight RCTs with 747 elderly T2D patients, demonstrated that exercise interventions significantly improved cognitive function compared with controls (*S* *M* *D* = 0.65, 95% CI 0.48–0.82, *p* < 0.00001; *I* ^2^ = 69*%*). Subgroup analyses revealed mean improvements of +2.22 points on the MoCA (95% CI 1.26–3.18) and +1.81 points on the MMSE (95% CI 0.71–2.90). Benefits were observed across different durations, with 3‐month programs yielding *M* *D* = 3.14 (95% CI 2.50–3.78), 6‐month programs yielding *S* *M* *D* = 0.32 (95% CI 0.12–0.52), and interventions longer than 6 months achieving *S* *M* *D* = 0.21 (95% CI 0.45–0.81). Both single‐mode and multimodal exercise produced cognitive gains, though the effect was greatest with multimodal training (*S* *M* *D* ≈ 0.86, 95% CI 0.39–1.33, *p* < 0.0001) [85].

In conclusion, a substantial body of evidence supports the role of physical exercise—particularly aerobic, resistance, and multimodal training—in alleviating cognitive decline associated with diabetes. These benefits are primarily mediated through improved insulin sensitivity, reduced neuroinflammation, enhanced neuroplasticity, and preservation of brain structure and function.

### 3.2. The Effects of Exercise Against DPN

The epidemiological rise of diabetes and prediabetes has been accompanied by an ascending rate of diabetic complications, with neuropathy being the most prevalent complication. Diabetic neuropathy, happening in almost 50% of DM patients, is identified as a set of symptoms, such as sensory loss and pain, occurring as a result of diffuse or focal damage to the peripheral nervous system and ANS [3, 86]. Diabetic neuropathy can appear in several forms, the most common being distal symmetric polyneuropathy, mostly represented in lower limbs and hands. Autonomic neuropathies, such as CAN, affecting sympathetic and parasympathetic nerves, are other types of diffuse neuropathies arising subsequent to diabetes [3, 4].

Diabetes duration and HbA1c levels are known to be significant predicting factors regarding diabetic neuropathy. Due to the complex mechanisms involved in DPN, its pathogenesis is not fully recognized, yet. However, several mechanisms are confirmed to participate in the DM‐associated neurodegeneration. Metabolic factors associated with DM can lead to increased oxidative stress and production of ROS, inflammation and release of inflammatory cytokines (i.e., TNF‐*α*, IL‐1*β*, and IL‐2), higher production of advanced glycation end‐products (AGEs), and DNA damage, eventually leading to endoneural vascular dysfunction, neuronal stress, and injury. DM‐associated microvascular alterations might play a crucial role in the pathophysiology of DPN, potentially via disruption of the blood‐nerve barrier and hypoxia [87, 88].

Diabetic neuropathy is associated with presentations such as pain, tingling, loss of sensation, and weakness, with a distal to proximal pattern of progression. NCV abnormalities in lower limbs can further solidify the proof of the presence of neuropathy. However, the diagnosis of diabetic neuropathy is mostly dependent on clinical features, as well as the history and examination of the patients. Diabetic neuropathy mostly represents itself via a loss of sensation to multiple sensory stimuli including a sharp object in the pinprick test, temperature variations, tuning fork vibration, or passive movement of the distal interphalangeal joint of the first toe (targeting proprioception sense) [3, 4, 89]. Furthermore, there are some unified scoring systems for diabetic neuropathy, merging clinical signs and symptoms of the disease, to provide a clear score and cut‐off values, aiding the diagnosis (i.e., the MDNS and the Toronto Clinical Neuropathy Score) [90, 91].

Diabetic neuropathy, as a disabling neurodegenerative disease, is highly related to diabetes‐associated falls, the risk of ulcerations, and limb amputations. Developing among 10%–20% of DM patients, diabetic neuropathic pain is another serious concern, representing itself through electric, burning, or stabbing sensations, often leading to hyperalgesia and allodynia [92, 93].

At present, glycemic control is considered the only definitive treatment option for diabetic neuropathy, with the efficacy of this therapy being more highlighted in T1D rather than T2D. In addition, multifactorial interventions including lifestyle modifications, physical exercise training, and weight loss are considered milestones of DPN management, particularly in the setting of T2D [87, 93].

Historically, individuals diagnosed with DPN were often advised to refrain from weight‐bearing activities, such as treadmill training, due to concerns regarding the potential risk of Charcot‐Marie‐Tooth joint deformities, skin breakdown, and subsequent foot infections [94]. In contrast, multiple studies have challenged this traditional advice, stating that weight‐bearing exercises not only are not associated with an increased risk of foot ulcers but also might have a protective effect upon foot skin [95–97].

In a randomized trial of aerobic cycling (3×/week, 12 weeks; *n* = 31), MDNS decreased from 12.2 ± 3.9 at baseline to 7.9 ± 2.4 postintervention, with improvements in HbA1c and leg perfusion. MDNS changes correlated with HbA1c reduction, which supports exercise as a low‐risk disease‐modifying strategy in DPN [98]. Furthermore, according to Zaccaria et al., statistical analysis and *χ* ^2^ test upon 90 T1D patients undergoing low, moderate, and high physical activity showed that DPN was significantly less prevalent among patients in the moderate to vigorous physical exercise group, according to Toronto consensus diagnostic criteria. Physical activity for at least 600 MET minutes per week was also suggested to be a protective factor concerning DPN [99]. Additionally, analysis of the data from 18,092 T2D patients from the UK Biobank in a cohort study implicated that any level of physical activity was accompanied by a lower risk of diabetic neuropathy. The minimal effective level of physical activity was a threshold of 1.5 h per week of walking [100]. Furthermore, human studies investigating nerve conduction velocity (NCV) have also confirmed that aerobic exercises could significantly increase NCV in peripheral nerves [101–103].

In addition to its physical health benefits, aerobic exercise has been shown to enhance the quality of life specific to neuropathy. An 8‐week regimen of moderate‐intensity exercise resulted in a decrease in Neuropathy Quality of Life (NQOL) scores. This questionnaire assesses patients′ subjective evaluations of functioning and quality of life across six specific domains, including restricted activity of daily living (RADL), disruptions in social relationships, and sensory–motor symptoms. These improvements in quality of life may be correlated with the positive effects observed in NCV studies [101].

The findings of preclinical studies suggest that aerobic exercises can mitigate the pathological processes underlying DPN. For instance, Ghaderpour et al. conducted a study demonstrating that 10 weeks of voluntary treadmill physical activity reduced allodynia in the von Frey filament test. Regular daily physical activity also prevented hyposensitivity, a condition observed in the late stages of diabetic neuropathy in rats. Beyond alleviating the animals′ sense of pain, aerobic exercises led to a reduction in the sciatic nerve axonal diameter and *G*‐ratio (the ratio of axon diameter to the diameter of the nerve fiber which represents the myelin thickness), among active diabetic rats [104].

In contrast, another study in mice found that diabetes induction resulted in thermal hyperalgesia after 18 weeks. However, this study revealed that low and moderate‐intensity aerobic exercise delayed responses to thermal stimulation and reduced mechanical sensitivity, with moderate‐intensity exercise demonstrating a more significant reduction in mechanical sensitivity [105].

Further histological investigations regarding the role of aerobic exercise over changes in the cellular structure of L5 dorsal root ganglia (DRG) have shown improvements in diabetic‐induced alterations among A cells of DRG, subsequent to 10 weeks of treadmill training. These cells are involved in diabetic‐induced vibratory sensation dysfunction. Aerobic exercise increased the cellular and nuclear volumes of A cells, mitigating the diabetes‐induced changes in cellular distribution. However, training was ineffective in reducing diabetes‐associated pathological reduction of cellular and nuclear volume of B cells [106]. Further research has indicated that 4 weeks of swimming exercise can mitigate diabetic‐induced reduction of IENFD [107]. IENFD is a validated marker for assessing peripheral distal neuropathy in both humans and rats [108, 109]. However, it should be noted that excessive walking has been shown to increase mechanical allodynia and reduce IENFD among diabetic rats [110].

Parallel to the aerobic exercises, resistance training was also investigated in different studies, in regard to its effect on diabetic neuropathy. In a controlled trial conducted by Gholami et al., a 12‐week program of moderate‐intensity resistance training resulted in a significant increase in neuropathic symptoms and improvements in sensory and motor nerve conduction velocities [111]. However, it is essential to mention that the same 12‐week controlled trial did not demonstrate any significant increase in IENFD, possibly due to limited follow‐up to observe alterations in neuron structures [112].

Furthermore, there has been growing interest in the combined resistance and aerobic exercise interventions, as it was recommended by organizations such as the American Sports Medicine Association, the Exercise Association of Australia, and the Belgian Physical Therapy Association [113]. It was concluded by some studies that combined exercises may be more effective in controlling HbA1c among diabetic patients [114]. Some studies have focused on the combined intervention for the management of diabetic neuropathies. A 10‐week combined exercise pretest posttest trial, involving 17 patients with DPN, showed promise in alleviating subjective pain and neuropathic pain assessed by the MNSI, as well as improving proximal IENFD measures. However, the NCV study and quantitative sensory testing following the exercise did not show significant changes [115]. Conversely, a controlled trial conducted by Beigi et al. suggested that 10 weeks of combined aerobic and unilateral lower extremity exercise improved NCV in the sural sensory nerve and peroneal motor nerves [113]. The findings from a recent double‐blinded RCT on T2D patients and DPN, with a mean age of 60.80 ± 9.73, who underwent structured strength and balance training, as well as aerobic training for 3 times a week for 8 weeks, indicated that both forms of exercise led to improvement of quality of life and DPN severity according to the Toronto clinical neuropathy system. There was no significant difference found between the two groups regarding these measures [116]. Some studies have specifically attempted to compare the effects of aerobic and resistance training in patients with diabetic distal symmetric polyneuropathy; however, they found no significant beneficial effect of exercise regardless of its type on NCV [47]. Additionally, a systematic review and meta‐analysis performed on 11 RCTs with 517 participants showed that the quality of evidence supporting the beneficial short‐term effects of exercise concerning neuropathic signs and symptoms in diabetic neuropathy is very low [117]. Therefore, further research is required in this field.

### 3.3. The Protective Role of Exercise Against CAN

CAN is characterized by the disruption of cardiovascular autonomic control, which is linked to a higher risk of morbidities associated with cardiac arrhythmia, silent ischemia, and MI. CAN is represented by the loss of HRV in the early stages of disease, which later progresses into more prominent symptoms including resting tachyarrhythmia, orthostatic hypotension, and tach/bradycardia [18]. Some studies have delved into the investigation of the role of exercise interventions against diabetes‐associated ANS dysfunction, specifically CAN.

As detailed in a systematic review and meta‐analysis of 21 studies with a total of 523 T2D participants, 472 of whom were undergoing exercise training, conducted by Picard et al., aerobic exercise has demonstrated significant effectiveness in enhancing HRV among individuals with T2D. This enhancement is attributable to both an increase in parasympathetic tone and a decrease in sympathetic tone [118]. However, it is noteworthy that fewer studies have explored the effects of resistance training on CAN [118]. A recent controlled trial that evaluated the resistance training outcomes confirmed its beneficial impact upon cardiac autonomic control parameters over a 12‐week period, among T2D individuals [119]. Some studies aimed to compare the role of resistance and aerobic training against CAN. In a study led by Moawd et al., the outcomes of resistance training and aerobic training were compared among diabetic patients with CAN. This study revealed that both types of exercises, for a duration of 3 months, yielded positive outcomes in terms of improving HRV parameters. Nevertheless, in certain instances, the effects of aerobic exercise appeared to be more pronounced [120]. Conversely, the results of another study by Bellavere et al. indicated that following 4 months of either aerobic or resistance training, similar improvements were observed in HRV indices of patients with CAN [121]. In addition, a systematic review and meta‐analysis comparing various types of exercise interventions, regarding their effectiveness in the improvement of CAN, concluded that endurance exercise exhibited the most substantial benefits in terms of HRV parameters compared to high‐intensity interval training (HIIT) and resistance training. It is essential to mention that the majority of studies included in this systematic review primarily focused on the effects of aerobic exercise, which may explain its perceived superiority [118]. In a RCT study carried out by Michou et al. on 25 kidney transplant recipient T2D participants with CAN, they demonstrated the improvement of functional capacity and cardiac autonomic function, subsequent to a 6‐month home‐based combined (aerobic and strengthening) training program, as indicated by higher VO2peak and 30‐s STS, as well as elevated SDNN and rMSSD among exercising patients compared to the controls [122].

Considering the current dearth of evidence regarding the role of resistance training and its comparative efficacy to other exercise interventions regarding CAN, it is advisable for future studies to prioritize comparative study designs to provide a more comprehensive understanding of their respective impacts.

## 4. Irisin and Physical Training: The Protective Role of This Exercise‐Induced Myokine Against Diabetes Complications

### 4.1. Irisin: Generation, Molecular Structure, and Function

Irisin, a myokine produced in the process of physical exercise, is initially derived from its precursor FNDC5, a muscle cell–expressed transmembrane protein regulated by peroxisome proliferator‐activated receptor‐*γ* coactivator 1*α* (PGC‐1*α*) [42].

FNDC5, first discovered in 2002, mostly distributed in muscle, adipose tissue, brain, and liver consists of 212 amino acids in humans containing the following main structural components: an (N)‐terminal signaling domain, a type III fibronectin domain, a hydrophobic component, and a cytoplasmic (C)‐terminal [123, 124]. Irisin, a molecule produced subsequent to proteolytic cleavage of FNDC5, was first discovered in 2012. Irisin has a crystal structure containing a fibronectin type III domain making up an intersubunit 8‐strand *β*‐sheet dimer structure attached to the flexible (C)‐terminal. The linkage between two domains is strengthened by hydrophobic and van der Waals forces [125, 126]. Irisin contains 112 amino acids with a total molecular weight ranging from 20 to 32 kDa [127]. The structure of the irisin hormone is highly preserved among all mammalian species, as it is evident by 100% structural identicalness between humans and mice [27].

As both a myokine and adipokine, irisin has been proven to be a crucial factor in thermogenesis and the regulation of energy expenditure by converting white adipose tissue into beige adipose tissue. This process is associated with antiobesity and antidiabetic effects through the expression of mitochondrial uncoupling protein 1 (UCP1) and plays its biological role via mitogen‐activated protein kinases (MAPKs) [27, 128–130].

### 4.2. Irisin and Physical Training

The beneficial effects of exercise, particularly concerning the enhancement of mental health and cognitive function in the context of DM, have been extensively studied. As irisin is released during physical exercise, it is believed to play a significant role in the protective effects of exercise on the mental and cardiovascular health of DM patients [131]. Acute elevation of irisin levels followed by a bout of physical training is supported by numerous studies, as it was confirmed by a meta‐analysis of 10 studies, reporting that acute bouts of exercise were accompanied by an average increment of irisin concentration of 15.0 postexercise among adults [132, 133]. The exercise‐induced increase of irisin was more remarkable in the setting of diabetes and prediabetes or subsequent to the application of resistance and combined types of training [134]. Chronic exercise (≥ 8 weeks) has also been shown to significantly reduce irisin levels in RCTs [135]. Interestingly, the acute rise in irisin levels appears to be independent of training status, although it may be associated with the type of training performed [136]. In a controlled human study, Żebrowska et al. [137] demonstrated that in adults with T1D, a single session of moderate‐intensity cycling under normoxic conditions significantly increased serum BDNF and total IGF‐1 levels. Notably, when the same exercise was performed under hypoxic conditions (FiO_2_ = 15*%*), it led to a greater and more sustained increase in free IGF‐1 and irisin levels. These changes persisted up to 24 h postexercise and were accompanied by improved glycemic profiles, suggesting a synergistic effect of hypoxia and exercise in modulating neurotrophic and metabolic markers relevant to diabetic health.

Furthermore, increases in irisin were observed following HIIT among youth with healthy weight, with no significant differences in obese or overweight participants [138]. Irisin levels peaked higher after HIIT compared to moderate‐intensity continuous training (MICT) [138]. It has been suggested that irisin secretion is more elevated following high‐intensity exercises compared to low‐intensity exercises, even when energy expenditure is matched [139]. In a study by Molnár et al. [140], they investigated the hormonal effects of a 6‐week aerobic exercise program consisting of treadmill and cycling, in 30 adults with T2D and sensory polyneuropathy. While the intervention led to notable metabolic improvements—including reductions in BMI (from 31.6 to 31.0 kg/m^2^), HbA1c (from 7.09% to 6.78%), and TNF‐*α* (from 0.70 to 0.57 pg/mL)—and significantly increased serum FGF‐21 levels (median 140.6–168.9 pg/mL), circulating irisin levels remained unchanged. This differential hormonal response suggests that irisin may not uniformly respond to aerobic training in all diabetic populations or under all protocols.

**Figure 2:**
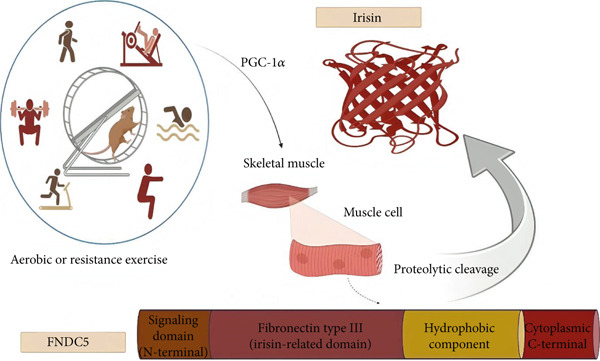
A graphical summary of the production of irisin from FNDC5 cleavage in the process of aerobic and resistance exercise.

Resistance exercise training has also been confirmed to increase irisin levels, as well as act as an effective intervention in preventing aging‐induced muscle function deterioration (Figure 2) [141]. Li et al. in a study to evaluate the effectiveness of different types of exercise on myocardial expression of irisin reported better results of cardiac function improvement in irisin‐mediated resistance training, via activation of the irisin/FNDC5‐PINK1/Parkin‐LC3/P62 pathway on a rat model of myocardial infarction. In this study, however, all forms of exercises and skeletal muscle electrical stimulation led to significant reduction of left ventricle internal dimension diastole and systole (LVIDd and LVIDs), as well as an increment of ejection fraction (EF) and fractional shortening (FS); these changes were more prominent in resistance training [142]. Furthermore, in their study of subjecting C57BL/6 mice to aerobic and anaerobic electrical pulse stimulation (EPS), Lavi et al. [143] confirmed that expression of FNDC5 was dependent on the type of activity and muscle fibers, as their data pointed toward increased FNDC5 and Pgc‐1*α* expression levels in all muscle types of the training group compared to sedentary controls, accompanied by a more significant increment in soleus muscle with a dominance of slow‐twitch fibers compared to other muscles mostly composed of fast fibers. Additionally, elevation of FNDC5 expression subsequent to both aerobic and anaerobic EPS was meaningfully higher in soleus muscle compared to extensor digitorum longus containing mostly fast fibers subsequent to EPS. Amuri et al. performed a RCT study in which 32 obese adults underwent 12 weeks of aerobic training allocated into MICT and HIIT groups. As a result, circulating irisin declined equally in both training groups with a more significant reduction in the male population of the study [144]. To determine whether different exercise intensities produce varying levels of irisin, Arabzadeh et al. [145] conducted an experiment where rats with induced T2D underwent moderate‐intensity interval training (MIIT) and HIIT. The results indicated a decrease in myoblast markers and a correlated reduction in FNDC5 mRNA expression in diabetic rats compared to healthy controls. Additionally, MIIT was more effective in promoting myogenic differentiation and elevating FNDC5 and myogenin levels than HIIT in diabetic rats. In a comprehensive study, Kurdiova et al. aimed to elucidate the tissue‐specific and complex mechanism of action of irisin. A positive correlation was confirmed between irisin and metabolic health, based on increased muscle mass and improved strength and contractility during habitual physical activity, along with increased circulating irisin levels [146]. Moreover, there is evidence supporting the effectiveness of aerobic exercise in increasing irisin levels and improving cognitive performance and depression in patients with multiple sclerosis [147].

### 4.3. Irisin and Diabetes

There is an increasing body of evidence suggesting the protective role of irisin in cardiovascular disorders, obesity, and more importantly diabetes. Several studies also confirmed the improvement of insulin resistance in the setting of FNDC5/irisin overexpression, in addition to irisin and insulin resistance index being positively correlated [148–150]. The reduction of irisin levels in T2D patients is supported by most studies; this reduction is also more significant when T2D is accompanied by its complications regardless of treatment or duration of the disease [146, 151, 152]. Additionally, according to an experiment conducted by Yang et al., a reduction of adipose tissue irisin level was observed in high‐fat diet (HFD)–fed obese mice which could be associated with skeletal muscle insulin resistance [153]. Moreover, lower irisin levels were observed in newly diagnosed cases of diabetes, gestational diabetes, and diabetic patients with renal insufficiency [154]. Furthermore, a study by Berezin et al. confirmed a link between irisin levels and glycemic control in diabetes, as irisin levels significantly decreased in poor glycemic control cases (HbA1c ≥ 7.0*%*) among T2D patients with heart failure [155]. In a study on mice with T2D, subsequent to persistent subcutaneous perfusion of irisin, improvement of glucose homoeostasis was observed in the form of alleviation of insulin resistance, gluconeogenesis reduction, and increase of glycogenesis, along with the rise of irisin levels [156]. Additionally, a study by Zheng et al. [157] proposed irisin as a novel therapeutic agent for T2D due to its role in amelioration of lipotoxicity‐induced *β* cell‐associated insulin resistance affecting phosphoinositide 3‐kinase (PI3K)/protein kinase B (AKT) [PI3K/AKT] insulin signaling pathway on HFD‐fed mice.

### 4.4. Irisin and Its Possible Role in Diabetic Cognitive Dysfunction

There are multiple studies implicating the involvement of irisin in neuroprotection and the regulatory role of exercise‐associated expression of brain FNDC5/irisin signaling pathway in cognition, with irisin being a critical regulatory component of exercise‐induced cognitive improvement [31–33]. Table 2 displays the features and findings of key studies to date examining the potential protective role of irisin against neuroinflammation, cognitive impairment, and neuropathies associated with DM. In furthering the discussion across these topics, it is worthwhile to mention the therapeutic properties of irisin in the treatment of cerebral ischemia reperfusion injury, which was characterized by improvement of brain cells′ apoptosis, along with reversal of biochemical changes, both in vivo and in vitro [173]. Lourenco et al. in a study on a mouse model of Alzheimer′s disease (AD) confirmed FNDC5/irisin to play a key role in the exercise‐mediated improvement of synaptic failure and memory impairment in AD [42]. Increment of irisin in hippocampal cells and a subsequent rise of BDNF followed by exercise can be indicative of neuroprotective and anti‐inflammatory properties [33]. The results of a study by Wrann et al. [41] indicated that in the process of endurance exercise, the BDNF‐associated cognitive function improvement, mostly affecting the hippocampus and dentate gyrus, could be regulated by the PGC‐1*α*/FNDC5/BDNF pathway. The levels of BDNF were, respectively, increased and decreased during forced expression and knockdown of FNDC5 expression. Furthermore, FNDC5 can be expressed in the brain tissue, as well, with its knockdown being responsible for interfering with the maturation of neurons. This study in general confirmed the correlation of activation of PGC‐1*α* by FNDC5 during a 30‐day free wheel running exercise in mice to the production of BDNF as an important factor of brain development. Additionally, in a cross‐sectional study on elderly women, subsequent to a 16‐week aquarobic exercise program, significant increment of irisin and BDNF levels was observed which could account for the prevention of neurodegeneration and brain function enhancement [174].

**Table 2:** A summary of studies evaluating the potential protective role of FNDC5/irisin against neuroinflammation, cognitive impairment, and neuropathies associated with DM among clinical and preclinical studies.

**List**	**Study design**	**Subjects**	**Irisin/FNDC5 elevation method**	**Sample type**	**Detection method**	**Kit/antibody brand**	**Tests to assess diabetic cognitive impairment or neuropathy**	**Key findings**
Human studies								
Lin et al. [158]	Cross‐sectional study	133 Chinese T2D patients (T2DM for > 3 years)	None	Plasma	ELISA	ELISA kit, Cusabio, Wuhan, China	MoCA, Mini MMSE, digit span test, verbal fluency test, clock drawing test, logical memory test, auditory verbal learning test, and trail making tests A and B (TMT‐A and TMT‐B)	• Diabetic patients with mild cognitive impairment (MCI) show elevated plasma irisin levels compared to those with normal cognition, suggesting a link between higher irisin and cognitive disruption, particularly in executive function. Significantly higher irisin levels in the MCI group compared to the control group (a mean of 241.48 [145.208–564.815] compared to 139.86 [82.845–500.413]) • Higher HbA1c and irisin measures were independent factors of diabetic MCI, therefore may serve as positive prognostic markers for the condition
Żebrowska et al. [137]	RCT	14 T1D individuals and 14 healthy controls	Moderate‐intensity continuous exercise for 40 min at 50% lactate threshold in hypoxia (FiO_2_ = 15.1*%*) and normoxia (FiO_2_ = 20.9*%*)	Serum samples	ELISA for BDNF, IGF‐1, IGF‐1 binding protein‐3 (IGFBP‐3), and irisin	Biovendor (for irisin), DIAsource, Belgium (for IGF‐1, IGFBP‐3), Immuniq, Poland (for BDNF)	No cognitive assessment tests, only BDNF level evaluation	Compared to healthy controls, T1D patients had notably lower BDNF and total IGF‐1 and higher irisin serum levels, pre‐exercise. Exercise significantly elevated the BDNF and total IGF‐1 in T1D patients. Additionally, free IGF‐1 and irisin levels were significantly higher subsequent to exercise in hypoxia (irisin levels remarkably higher among T1D patients than the control group). In hypoxia, postexercise BDNF and total IGF‐1 levels in both groups showed positive correlations
Cokar et al. [159]	Prospective, single‐arm interventional study	16 female T2D patients, aged 40–65	Aerobic exercise on treadmill (40 min/day, at 50%–70% of the maximal heart rate, 3 days a week for 12 weeks)	Serum samples	ELISA for BDNF, nerve growth factor (NGF), and irisin	Abbkine, Wuhan, China (for irisin, NGF, and BDNF)	TMT‐A and TMT‐B for cognitive function, Beck Depression Inventory (BDI) for depression levels	No significant changes in BDNF (*p* = 0.271), NGF (*p* = 0.230), or irisin (*p* = 0.101) levels were found postexercise. Aerobic exercise improved TMT‐A (*p* = 0.001) and TMT‐B (*p* = 0.002) scores and decreased BDI scores (*p* = 0.031). Oxidative stress was improved, despite no significant change in neurotrophic/metabotropic factors. Irisin levels increased from 5.2 ± 1.3 ng/mL pre‐exercise to 5.4 ± 1.5 ng/mL postexercise
Li et al. [160]	A single center cross‐sectional study	108 patients diagnosed with T2DM (48 patients with MCI, 60 patients without MCI) and 23 normal controls	None	Serum samples for assessment of IL‐18 and irisin	ELISA	Not specified	MoCA and MMSE	• T2D patients showed significant cognitive impairment in MMSE and MoCA test compared to controls. The cognitive decline was more notable among the diabetic MCI group • The positive correlation of irisin levels with MoCA and MMSE scores indicates the possible protective role of irisin against diabetic MCI • IL‐18 levels were markedly increased in T2D patients compared to controls. This elevation was more significant among T2D patients with MCI compared to both study groups
Ghodrati et al. [161]	RCT	21 T2D elderly women (*n* = 12 undergoing training and *n* = 9 participants undergoing no exercise interventions as the control group)	An exercise regimen combining aerobic, resistance, and balance training for 3 times per week for 12 weeks	Serum samples for assessment of BDNF and irisin	ELISA	ELISA kits, Hangzhou East Biopharm, Hangzhou, China	MoCA, forward digit span test (for working memory), digit symbol substitution test (for processing speed)	• In addition to notable reduction of FBS and HbA1c levels and improvement of cardiorespiratory fitness, exercising participants also showed significant improvements regarding MoCA scores. However, no significant changes were observed in forward digit span and digit symbol substitution test results • After 12 weeks of combined exercise, no significant changes in BDNF and irisin levels were found in the exercise group (despite the significant decrease in irisin levels in the control group). Irisin levels in the exercise group were 5.4 ± 3.1 ng/mL before and 4.4 ± 3.0 ng/mL after exercise
Haddad et al. [162]	Cross‐sectional study	‐60 T2D patients, 19 of them with DPN (31.6%) ‐30 healthy controls	None	Serum samples	ELISA	Irisin ELISA Kit (Wuhan EIAab Science Co. Ltd., China)	‐Semmes‐Weinstein monofilament examination and 128 Hz ‐Tuning fork for diagnosis of DPN	• However, there was not a significant difference between irisin levels of diabetic patients and healthy population (0.18 ± 0.10 irisin levels among the DM group compared to 0.16 ± 0.05 among controls), the DPN group had remarkably lower irisin levels compared to non‐DPN T2D patients (0.11 ± 0.05 vs. 0.22 ± 0.11 ng/mL, *p* < 0.001), therefore suggesting the potential microvascular complications due to the role of irisin reduction in endothelial dysfunction and inflammation • Irisin levels were negatively correlated with DM duration, FBS, HbA1c, and fasting insulin levels in DM patients
Molnár et al. [140]	Prospective cohort study	30 T2DM patients with distal sensory polyneuropathy and 32 age‐ and gender‐matched T2DM patients without neuropathy were controls	Aerobic exercise (treadmill and cycle ergometers 3 times a week for 70 min for 6 weeks)	Serum samples	ELISA for FGF21, TNF‐alpha, irisin, leptin, and adiponectin	Biovendor (for FGF21, irisin), R&D Systems (for TNF‐alpha, leptin, and adiponectin)	Neurometer for current perception threshold (CPT)	No significant change in serum irisin levels was found (pre‐exercise: 4.32 ± 1.33 * μ*g/mL, postexercise: 4.3 ± 1.29 * μ*g/mL). Aerobic exercise increased FGF21 levels and improved sensory neuropathy (CPT) in T2DM patients. Significant correlations were found between changes in FGF21 levels and the improvement of CPT, as well as changes in TNF‐alpha levels and BMI
Preclinical studies								
Yang et al. [163]	In vitro study (cell culture)	HT22 murine hippocampal cells	FNDC5 overexpression via plasmid transfection and exogenous irisin supplementation	HT22 cell culture	CCK‐8 assay for cell viability, FerroOrange staining for intracellular Fe2+, MDA assay, Western blot for synthetase long chain family member 4 (ACSL4), PSD95, FNDC5, and *β*‐actin	R&D Systems (for irisin)/Abcam (for FNDC5)/Santa Cruz Biotechnology (for ACSL4)	Not applicable	High glucose induced neurotoxicity and ferroptosis in HT22 cells. FNDC5/irisin treatment mitigated neurotoxicity and ferroptosis by enhancing cellular antioxidant capacity, inhibiting ACSL4 expression, and improving intracellular redox status. Exogenous irisin supplementation also alleviated high glucose–induced neurotoxicity and ferroptosis in cells with reduced FNDC5 expression. These results support the neuroprotective role of FNDC5/irisin in diabetic neuropathy
Gamal et al. [164]	Experimental animal study	40 T2DM rats with neuropathy	Aerobic exercise (treadmill and bicycle ergometers, 3 times a week for 70 min)	Whole brain (for irisin receptors), gastrocnemius muscles and abdominal adipose tissue	Western blot for MAPK, PCR for gene expression (of irisin, irisin receptor, and BDNF)	Abcam, Cambridge, Massachusetts, United States (for BDNF, MAPK), Invitrogen (for FNDC5)	Spontaneous alternation test in T‐maze (cognitive performance measured by the number of correct alternations and time consumed per arm entry)	Chronic and acute exercise in diabetic rats increased muscle irisin and brain receptor expression, positively correlating with cognitive function (T‐maze) and improved insulin sensitivity. Significant correlations between muscle irisin levels and brain BDNF, MAPK, and cognitive function were observed. Both exercise regimens resulted in better glucose and lipid profiles. Chronic exercise was more effective than acute exercise in improving cognitive performance
Huang et al. [166]	Experimental animal study	STZ‐induced diabetic rats	Application of the irisin overexpression vectors	Serum samples and hippocampal tissue	ELISA for assessment of serum GHbA1c, advanced glycated end products (AGEs), BDNF, and irisin; immunohistochemical analysis for BDNF expression in hippocampal tissue	Rat BDNF ELISA kit (Cat. No. ml302829), rat irisin ELISA kit (Cat. No. ml0373721), rat GHbA1c ELISA kit (Cat. No. ml024079), and rat AGE ELISA kit (Cat. No. ml003305) from Shanghai Enzyme‐Linked Biotechnology Co. Ltd., China	No cognitive assessment tests, only BDNF level evaluation	• Animals receiving irisin overexpression vector showed significantly higher serum concentrations and hippocampal expression of irisin and BDNF, as well as reduced serum glycosylated hemoglobin A1c (GHbA1c) and AGE levels compared to DM rats not receiving any vectors or receiving irisin interference vector • Irisin treatment led to improvement of DM‐induced reduction of hippocampal cell viability, according to cell cultures
Wang et al. [68]	Experimental animal study	STZ‐induced diabetic Mice	I.p. administration of irisin	CSF samples	Western blot analysis for GFAP and synaptophysin (SYP) ELISA for IL‐6 and IL‐1*β* assessment	Western blot (GFAP) (1:4000; Millipore Corp., Massachusetts, United States), synaptophysin (SYP) (1:4000, Abcam, Cambridge, United Kingdom), ELISA kits (eBioscience, Thermo Fisher Scientific)	‐Y‐maze test for assessment of spatial working memory ‐Novel object recognition for assessment of nonspatial memory	• Irisin injection attenuated STZ‐induced impairment of short‐term spatial memory and spontaneous alternation and prevented STZ‐induced memory deficits • Irisin prevented the upregulation of GFAP expression in DM mice and alleviated the STZ‐induced reduced synaptophysin expression • Irisin reduced the elevated hippocampal and CSF measures of IL‐1*β* and IL‐6, in addition to increased P38, STAT3, and NF*κ*B hippocampal protein activation subsequent to DM
Lang et al. [167]	Experimental animal study	STZ‐induced C57BL/6J T2D mice	8‐week moderate‐intensity treadmill training	Hippocampus samples	Western blotting for proinflammatory cytokines (IL‐1*β*, IL‐6, and TNF‐*α*), anti‐inflammatory cytokines (IL‐10, TGF‐*β*1), SIRT1, NF‐*κ*B, FNDC5, PGC‐1*α*, BDNF; RT‐PCR analysis for total RNA extraction on hippocampus samples	FNDC5 (Proteintech, Wuhan, China), BDNF, TNF‐*α*, IL‐6, IL‐10, SIRT1, IL‐1*β*, and TGF‐*β*1 (ABclonal, Wuhan, China), NF‐*κ*B and PGC‐1*α* (Abcam, Cambridge, United Kingdom)	MWM test (for spatial learning and memory)	• Diabetic mice undergoing treadmill exercise showed increased activity in the SIRT1/PGC‐1*α*/FNDC5 signaling pathway and higher BDNF expression, along with inhibition of the SIRT1/NF‐*κ*B pathway • Long‐term exercise reversed significant DM‐induced disruptions in spatial memory and learning • Exercise reduced DM‐induced M1 microglia activation, the main source of proinflammatory cytokines, and improved neuroinflammation by decreasing IL‐1*β*, IL‐6, and TNF‐*α* levels while increasing IL‐10 and TGF‐*β*1 expression
Lee et al. [168]	Experimental animal study	STZ‐induced C57BL/6 T2D mice	Treatment with *Annona muricata* extract (AME)	Liver, gastrocnemius tissue, and hippocampus	Western blot analysis	Antibody reagent (Bio‐Rad, Hercules, California, United States)—PGC1*α* (sc‐517380), FNDC5 (ab131390), BDNF (ab108319)	No cognitive assessment tests, only BDNF level evaluation	• Increased release of myokines like BDNF, irisin, and CTSB after AME treatment may positively impact brain metabolism and physiology due to their roles in neurogenesis and angiogenesis • Elevated PGC‐1*α* activity from AME prevents neurotoxic KYN accumulation in the brain by converting it into neuroprotective kynurenic acid (KYNA), thereby providing hippocampal neuroprotection, as indicated by the restoration of CTSB and BDNF levels reduced by diabetes
Xiang et al. [169]	Experimental animal study	Diabetic db−/− mice with cognitive impairment (CI) and normal wild‐type (WT) mice	FNDC5 overexpression via gene transfection	Hippocampal tissue	Single‐cell RNA sequencing (scRNA‐seq), real‐time PCR, Western blot	Not specified	TMT, cognitive assessment based on neuronal synaptic plasticity and gene expression levels	• Overexpression of FNDC5 in hippocampal cells increased BDNF and synapsin‐1 expression, enhancing synaptic plasticity • Hyperglycemia‐related downregulation of FNDC5 was associated with altered H3K4/H3K9 methylation at its promoter • FNDC5/BDNF‐Trk axis may exert neuroprotective effects in hyperglycemia‐induced cognitive impairment by modulating neuron–glia communication
Xu et al. [171]	Experimental animal study	T2DM mice	10 weeks of aerobic exercise (treadmill training)	Brain samples (to obtain hippocampal tissue)	Western blot analysis	Rabbit anti‐FNDC5 (1:1000, A18107, ABclonal, Wuhan, China) Rabbit anti‐TLR4 (1:500, WL00196, Wanleibio, LN, China) Mouse anti‐NF*κ*B (1:1000, sc‐8008, Santa Cruz, California, United States)	MWM test	Exercise significantly increased (FNDC5)/irisin protein levels, in addition to notable reduction of TLR4 and NF‐*κ*B hippocampal expression of T2DM mice. However, among these mice, blocking irisin receptor signaling led to downregulation of TLR4/MyD88/NF‐*κ*B Significant reduction of T2DM‐induced inflammation and notable promotion of hippocampal neurogenesis and memory function was observed postexercise (via irisin/TLR4/MyD88/NF‐*κ*B signaling pathway activity). Adversely, 10 weeks of treatment with cyclo RGDyk, an irisin receptor signaling inhibitor, reversed these positive outcomes
Hassan et al. [172]	Experimental animal study	STZ‐induced diabetic rats	Irisin treatment for 8 weeks	Sciatic nerve tissue and serum samples	ELISA for TNF‐*α*; GPX activity (UV method); TBARS assay (colorimetric method)	TNF‐*α* ELISA kit (Catalog No. K0331196) (KOMA BIOTEC., Seoul, Korea), GPx kit (Cayman Chemical, United States)	‐Hot plate analgesia meter test ‐NCV of the sciatic nerve (its reduction confirming diabetic neuropathy)	• The mean NCV measures were higher in irisin‐treated T2D rats compared to healthy controls and diabetic rats without irisin treatment • Irisin treatment significantly reduced diabetes‐induced elevation of TNF‐*α* measures and sciatic tissue TBARS, in addition to significant increase of declined GPx measures
Kalkan et al. [34]	Experimental animal study	STZ‐induced diabetic rats	Low and high‐intensity exercise (LIE and HE) for 8 weeks; with acute irisin injection (at doses of 1, 10, and 20 micg/kg at the end of the 8th week at three of the groups)	Serum samples	ELISA	ELISA kit (Sunlong Biotech, Cat No. SL0827Ra, People′s Republic of China)	‐Mechanical induced withdrawal threshold ‐Radiant heat induced withdrawal threshold	• Diabetic rats exhibited increased thermal pain thresholds compared to healthy controls at 4 and 6 weeks, but by the 8th week, the exercise groups showed a significant decrease in thermal thresholds compared to other diabetic rats • While diabetes increased pain thresholds at 6 and 8 weeks, the LIE and HIE groups had significantly lower pain thresholds compared to other diabetic rats • Diabetic rats had lower irisin levels than controls after 8 weeks, but the LIE and HIE groups had higher irisin levels compared to both controls and nonexercising diabetic groups • Following 60 min, acute irisin administration reduced thermal pain thresholds at all doses compared to controls

Irisin blood level has been investigated as a marker of cognitive function in recent studies in health and disease conditions. Lan et al. [175] found a positive correlation between the Stroop Color‐Word test (as an assessment tool for inhibitory control) and plasma irisin level among obese/overweight children in a cross‐sectional study with a total of 502 children aged 7–12. However, the irisin association with executive function had a significant interaction with body weight status. Consequently, irisin plasma level in children with normal or less than normal weight was not associated with executive function. Moreover, in a clinical study on 133 Chinese T2D patients, Lin et al. separated diabetic patients with MCI from healthy cognition controls, using the MoCA score. The data from this study pointed toward a correlation between higher plasma irisin level and poor executive function, along with higher irisin concentrations in T2D patients with MCI. They found an adjusted *β* of −0.002, 95% CI: −0.004 to 0, *p* = 0.01, for the association of irisin plasma level and MoCA score among the study participants [158]. In a cross‐sectional study by Li et al. [160], on 108 hospitalized T2D patients categorized based on the presence (36 patients) or absence of MCI (41 patients) and 18 controls, they found that irisin plasma level was significantly lower among diabetic patients with MCI (15.69 ng/mL, 95% CI: 4.71–31.61) compared to diabetic patients without MCI (32.81 ng/mL, 95% CI: 9.07–81.67) and compared to control individuals (116.22 ng/mL, 95% CI: 77.00–159.01). Moreover, they found a strong positive correlation with MoCA score (correlation coefficient = 0.724, *p* < 0.001) and MMSE (correlation coefficient = 0.456, *p* < 0.001) in their study population. This was also supported by a recent systematic review and meta‐analysis of 11 studies (*n* ≈ 1400), which demonstrated a modest but consistent positive correlation between circulating irisin levels and global cognition (pooled *r* = 0.26, 95% CI 0.10–0.41). Subgroup analyses confirmed similar associations across serum and CSF measurements as well as across different populations, and irisin was positively correlated with BDNF (*r* = 0.41), providing mechanistic plausibility [176].

Furthermore, the results of an RCT study by Ghodrati et al. [161], enrolling 21 elderly T2D women as study participants, showed that subsequent to 12 weeks of multimodal training, consisting of aerobic, resistance, and balance exercises, in addition to improvement of FBS and HbA1c levels, cardiorespiratory fitness, upper and lower body strength, cognitive function were enhanced among the training group, as well. Moreover, irisin levels which were significantly reduced among controls showed no significant changes among the training group. However, there were no significant changes in BDNF levels between the two groups. Furthermore, a single‐arm trial of aerobic exercise with a 1‐year follow‐up enrolled 16 female patients with T2D and assessed their irisin plasma levels and performance on the TMT for executive function at baseline, after one session, at 12 weeks, and at 1 year of exercise. They discovered that executive function improved, and the TMT score significantly declined over time. Nonetheless, the irisin level, despite fluctuations in plasma concentration, did not exhibit a statistically significant difference [159].

In addition to clinical evidence on the irisin‐cognitive function relation, preclinical studies have explored the mentioned association. In a recent study by Lee et al. [168], they aimed to examine the effects of *Annona muricata* extract (AME) on the improvement of multiorgan energy metabolism through the skeletal muscle–brain endocrine loop via measurement of associated myokines in T2D mice. They observed an improvement of cognitive impairment, a common consequence of T2D [177], simultaneous with an increase of irisin, as well as an elevation of other brain function–associated circulatory myokines, fibroblast growth factor 21 (FGF21), BDNF, and cathepsin‐B (CTSB), implicating the possible potential association of irisin on the improvement of brain function in diabetes. Furthermore, Gamal et al. [164] investigated the role of endogenous irisin in cognitive function and insulin sensitivity in a diabetic rat model exposed to either acute or chronic aerobic exercise. Both exercise interventions significantly upregulated muscle irisin expression compared to sedentary diabetic rats (*p* < 0.001). Muscle irisin mRNA levels showed strong positive correlations with brain irisin receptor expression, BDNF, and brain MAPK expression (*r* = 0.878, 0.933, and 0.908, respectively; *p* < 0.001 for all). These molecular changes were accompanied by improved cognitive performance in the T‐maze, better insulin sensitivity, and favorable lipid profile alterations, suggesting the potential role of exercise‐induced irisin in the enhancement of cognition and metabolic status in diabetes through BDNF‐related MAPK signaling. Xiang et al. [169] explored the impact of chronic hyperglycemia on hippocampal synaptic plasticity and found that FNDC5 plays a central role in modulating cognitive impairment. Single‐cell sequencing in diabetic mice revealed disrupted neuron–glia interactions and reduced FNDC5 expression, partly due to promoter methylation imbalance. In vitro, FNDC5 overexpression significantly upregulated BDNF and synapsin‐1 under high‐glucose conditions, highlighting the importance of the FNDC5/BDNF‐Trk axis in preserving synaptic function during hyperglycemia‐induced neurodegeneration.

In a study by Wang et al., diabetic mice demonstrated cognitive and memory impairment in behavioral tests, along with neuroinflammation and elevation of interleukin‐6 (IL6), interleukin‐1*β* (IL‐1*β*), and GFAP, as well as reduction of synaptic protein expression. Amelioration of the aforementioned changes along with inhibition of P38, STAT3, and nuclear factor‐*κ*B (NF*κ*B) activation by the application of irisin in this study could be suggestive of the anti‐inflammatory properties of irisin on CNS in the setting of DM [68]. In addition, Xu et al. [171] observed a significant increase in doublecortin (DCX)‐positive cells as a marker of neurogenesis in the mouse hippocampus alongside better performance in the MWM test of memory in the 10‐week treadmill training diabetic group compared to T2D mice without training. Additionally, they found that blocking the irisin receptors using cyclo RGDyk resulted in significantly fewer DCX+ cells and memory performance in the MWM test. These findings suggest possible involvement of irisin regarding the beneficial effects of treadmill exercise on diabetic‐induced cognitive impairment mitigation. Furthermore, the involvement of the Sirtuin 1 (SIRT1)/PGC‐1*α*/FNDC5/BDNF signaling pathway in the attenuation of diabetes‐induced neuroinflammation was confirmed in a T2D C57BL/6J mouse model in a study by Lang et al. who reported alleviation of cognitive dysfunction followed by 8 weeks of treadmill training, in addition to reduction of IL‐1*β*, IL‐6, and TNF‐*α*, along with elevation of IL‐10 and transforming growth factor‐*β*1 (TGF‐*β*1) anti‐inflammatory cytokines and BDNF release improvement [167]. Moreover, a recent in vitro study investigated the effects of FNDC5/irisin on HT22 cells under conditions of high glucose–induced neurotoxicity, specifically focusing on ferroptosis in a hyperglycemic environment. The study found that both FNDC5 overexpression and the addition of exogenous irisin can reduce intracellular iron overload, ROS, ferroptosis, and neurotoxicity. Additionally, the neurotoxicity associated with reduced endogenous irisin due to FNDC5 knockdown in a hyperglycemic environment was mitigated by the administration of exogenous irisin [163].

Given the key factors linked to cognition in individuals with diabetes, a neurotrophic element essential for memory and learning through synaptic regulation and the preservation of neural integrity—primarily abundant in the cortex and hippocampus—and HbA1C, a well‐established independent glycemic risk factor for cognitive decline, Huang et al. explored the potential role of vectors promoting overexpression and inhibition of irisin in diabetic rats. Their findings demonstrated an enhancement in hippocampal neurons′ viability, next to the improvement of MCI among diabetic rats, subsequent to irisin administration, due to the positive and negative correlations of irisin levels with serum BDNF and HbA1C, respectively, all implicating the possible role of irisin in the treatment and prevention of cognitive impairment and diabetic dementia [166]. Considering the role of irisin in lowering A*β*‐induced production of IL‐1*β* and IL‐6 inflammatory factors, along with the regulation of cyclooxygenase‐2 (COX‐2), AKT, and NF*κ*B in an astrocyte‐based culture, in addition to the presence of a close association of astrocytes in memory and cognition deterioration in diabetes, Wang et al. proposed irisin as a therapeutic candidate to mitigate diabetes‐induced cognitive and memory deficits [69]. In summary, irisin has emerged as a promising neuroprotective myokine that may mediate many of the cognitive benefits associated with physical exercise in diabetes. Through its anti‐inflammatory, neurogenic, and synaptic effects—particularly in the hippocampus—it holds potential as both a biomarker and therapeutic target for diabetes‐associated cognitive impairment.

### 4.5. Irisin and DPN

There is evidence of the neuroprotective footprint of irisin in alleviation of sensory and motor neuropathy, as it has been proven effective in attenuation of burn‐induced neuropathy and subsequent paresthesia, pain sensitization, muscular atrophy, and neuronal apoptosis, through its antineuroinflammatory properties. Additionally, spinal irisin gene delivery also ameliorated burn‐induced demyelination of the sciatic nerve and muscular innervation [165, 178].

In a study by Dameni et al., acute intrathecal administration of irisin was proven effective in the reduction of peripheral neuropathic pain among male rats [179]. Farzad et al. [180] also confirmed the attenuation of neuropathic pain and rehabilitation, following 4 weeks of swimming training, the effects of which were considered to be partially associated with the expression of irisin.

The data from a study by Kalkan et al. demonstrated an increase in serum levels of irisin, accompanied by improvement in neuropathy in exercising diabetic rats compared to nonexercising ones, suggestive of the neuroprotective role of irisin during exercise in diabetes. Furthermore, these changes were more significant in high‐intensity compared to low‐intensity exercise. Pain threshold was also increased in diabetic rats receiving irisin injection [34]. Hassan et al. also designed a study in which they confirmed the alleviating role of irisin regarding NCV abnormalities in T2D‐associated diabetic polyneuropathy, in addition to improvement in sciatic tissue damage in male albino rats, due to the anti‐inflammatory and antioxidant properties of irisin. In this study, irisin levels were 40% lower in the diabetic group compared to the irisin‐treated group. Expression of sciatic nerve irisin receptors was also significantly increased subsequent to irisin treatment in diabetic rats [172].

Haddad et al. [162] also aimed to assess the effect of irisin, a previously recognized factor for muscular enhancement of glucose uptake and glucose transporter‐4 expression, on micro and macrovascular complications of T2D. DPN was evaluated as one of the complications. In this study, contrary to the previous ones, mean serum irisin was not significantly different in T2D patients compared to the control group; however, there was a significant negative correlation between mean irisin levels and glycemic indices such as fasting blood sugar, HbA1C, and fasting glucose. A remarkable decline in irisin levels was also observed in the DPN group compared to the corresponding non‐DPN group. Moreover, the results of a study by Xiang et al. [170] also pointed toward a tight correlation between circulating irisin levels and endothelial dysfunction in T2D. There is also evidence of endothelial function improvement using exogenous irisin via a decline in oxidative and nitrative stress [181].

## 5. Limitations and Future Directions

While this narrative review is aimed at providing a comprehensive overview of the effects of exercise and the myokine irisin on diabetes‐related cognitive impairment and neuropathies, several limitations must be acknowledged. A key challenge is the substantial heterogeneity across the included studies. These studies vary widely in terms of design—ranging from cross‐sectional analyses and RCTs to multiple types of preclinical animal experiments—as well as in population characteristics, including individuals with T2D and STZ‐ or HFD‐induced rodent models. Additionally, exercise interventions differ in modality (aerobic, resistance, or multimodal), intensity, frequency, and duration, making direct comparisons difficult and precluding quantitative synthesis. Although we attempted to address this issue by categorizing studies by exercise type and separating human from preclinical data in both the narrative and tables, methodological inconsistencies remain a significant limitation.

Another important consideration relates to the generalizability of findings. Most clinical trials included relatively small and often single‐center samples, frequently from specific ethnic or regional backgrounds, which limits the extrapolation of results to broader populations. In particular, older adults with advanced neuropathic complications or multiple comorbidities are often underrepresented. Similarly, most preclinical studies rely on male rodent models, restricting insights into potential sex‐related differences in exercise or irisin responses.

Potential biases should also be acknowledged. Lack of blinding in several trials raises the possibility of performance or detection bias, while publication bias may favor positive findings in the literature. Furthermore, heterogeneity in outcome measures—ranging from different cognitive test batteries to diverse indices of autonomic and peripheral nerve function—creates a risk of selective reporting bias, particularly when only statistically significant endpoints are emphasized. By reporting effect sizes and confidence intervals whenever available, we sought to mitigate this limitation.

Although aerobic training has received the most attention, the effects of resistance and especially multimodal exercise interventions remain underexplored. Emerging evidence suggests that combining exercise modalities may yield superior benefits in improving neurocognitive and neuropathic outcomes in diabetes. However, few studies have directly compared these approaches. Future large‐scale randomized trials should systematically evaluate different exercise modalities and their relative impact on cognitive function and neuropathy, ideally using harmonized protocols and rigorous outcome assessments. Moreover, these trials should be designed with adequate power, control for confounding variables, and adhere to established reporting standards such as CONSORT to improve reproducibility and comparability. Additionally, long‐term follow‐up is still scarce, making it unclear whether exercise‐ or irisin‐associated benefits are durable and translate into clinically meaningful outcomes such as reduced incidence of dementia or slower neuropathy progression.

Moreover, more studies are needed to determine whether changes in irisin levels following exercise are causally linked to cognitive or neuropathic improvements. Research that manipulates irisin signaling (e.g., through receptor antagonism, gene overexpression, or recombinant protein administration) in conjunction with exercise may help clarify its function. Furthermore, the potential of irisin as a biomarker or therapeutic adjunct in diabetes‐associated neurological complications should be explored in both preventive and interventional frameworks.

Finally, to synthesize the current body of research and guide evidence‐based practice, systematic reviews and meta‐analyses are warranted. These should be stratified by study design (clinical vs. preclinical), exercise type (aerobic, resistance, multimodal), and outcome domain (cognition vs. neuropathy), in order to determine effect sizes, explore heterogeneity, and identify moderators of treatment response. Such efforts will not only clarify the strength and consistency of available evidence but also inform the development of targeted interventions to address the neurological burden of diabetes more effectively.

## 6. Conclusion

This review highlights the converging evidence supporting the beneficial impact of physical exercise and the myokine irisin on diabetes‐associated cognitive decline and peripheral neuropathy. Exercise interventions, particularly when sustained and structured, promote neuroplasticity, attenuate neuroinflammation, and enhance central and peripheral nervous system integrity in diabetic states. Irisin has emerged as a biologically plausible mediator of these effects, given its ability to influence neuronal signaling, synaptic maintenance, and oxidative stress pathways. The integration of behavioral and molecular research offers a promising framework for understanding the neuroprotective mechanisms at play and paves the way for future translational applications. Together, these findings support the potential of nonpharmacological, exercise‐based strategies in mitigating the neurological burden of diabetes, with irisin serving as a candidate target for therapeutic development.

**Nomenclature**
A*β*
: *β*‐amyloid
ACSM: American College of Sports Medicine
AD: Alzheimer′s disease
AGEs: advanced glycation end‐products
AME: *Annona muricata* extract
ANS: autonomic nervous system
BDNF: brain‐derived neurotrophic factor
BRS: baroreflex sensitivity
CAN: cardiac autonomic neuropathy
COX‐2: cyclooxygenase‐2
CTSB: cathepsin‐B
DPN: diabetic in peripheral neuropathy
DLPFC: dorsolateral prefrontal cortex
DM: diabetes mellitus
DMICC: Diabetes Mellitus Interagency Coordinating Committee
DRG: dorsal root ganglia
FGF21: fibroblast growth factor 21
FNDC5: fibronectin type III domain‐containing protein 5
FPA: frontal polar
GFAP: glial fibrillary acidic protein
GPx: glutathione peroxidase
GSH: glutathione
HIFT: high intensity low volume
HIIT: high‐intensity interval training
HRV: heart rate variability
ICE: integrated concurrent exercise
IDF: International Diabetes Federation
IENFD: intraepidermal nerve fiber density
IGF‐1: insulin‐like growth factor 1
KYNA: kynurenic acid
MAPKs: mitogen‐activated protein kinases
MCI: mild cognitive impairment
MDNS: Michigan Diabetic Neuropathy Score
MICT: moderate‐intensity continuous training
MNSI: Michigan Neuropathy Screening Instrument
MoCA: Montreal Cognitive Assessment
MMSE: Mini‐Mental State Examination
MWM test: Morris Water Maze test
NCV: nerve conduction velocity
NF*κ*B: nuclear factor‐*κ*B
NIH: National Institutes of Health
NQOL: Neuropathy Quality of Life
OFC: orbitofrontal cortex
PGC‐1*α*
: peroxisome proliferator‐activated receptor‐*γ* coactivator 1*α*
PI3K/AKT: phosphoinositide 3‐kinase (PI3K)/protein kinase B (AKT)
RADL: restricted activity of daily living
RCT: randomized controlled trial
RNFL: retinal nerve fiber layer
ROS: reactive oxygen species
SIRT1: Sirtuin 1
T1D: Type 1 diabetes
T2D: Type 2 diabetes
TGF‐*β*1: transforming growth factor‐*β*1
TBARS: thiobarbituric acid reactive substances
UCP1: uncoupling protein

## Ethics Statement

The authors have nothing to report.

## Consent

Our manuscript does not include any individual person′s data in any form.

## Disclosure

Figures 1 and 2 are original, unpublished before, and created with http://BioRender.com. All the authors have approved the submitted version of the manuscript. All the authors have consented both to be personally accountable for their own contributions and to ensure that questions related to the accuracy or integrity of any part of the work are appropriately investigated, resolved, and the resolution documented in the literature.

## Conflicts of Interest

The authors declare no conflicts of interest.

## Author Contributions

Sepideh Poshtdar contributed in study concept and design, manuscript drafting, writing the original manuscript, table preparation, and critical manuscript revision for important intellectual content. Hosein Ataei‐Goujani contributed in study concept and design and writing the original manuscript. Amirali Ahrabi contributed in screening articles, figure preparation, writing the original manuscript, substantial revising and editing of the manuscript, and preparing the final draft.

## Funding

No funding was received for this manuscript.

## Data Availability

The datasets used during the current study are available from the corresponding author upon reasonable request.
